# How to describe disordered structures

**DOI:** 10.1038/srep23455

**Published:** 2016-04-11

**Authors:** Kengo Nishio, Takehide Miyazaki

**Affiliations:** 1 National Institute of Advanced Industrial Science and Technology (AIST), Central 2, Umezono 1-1-1, Tsukuba, Ibaraki 305-8568, Japan

## Abstract

Disordered structures such as liquids and glasses, grains and foams, galaxies, etc. are often represented as polyhedral tilings. Characterizing the associated polyhedral tiling is a promising strategy to understand the disordered structure. However, since a variety of polyhedra are arranged in complex ways, it is challenging to describe what polyhedra are tiled in what way. Here, to solve this problem, we create the theory of how the polyhedra are tiled. We first formulate an algorithm to convert a polyhedron into a codeword that instructs how to construct the polyhedron from its building-block polygons. By generalizing the method to polyhedral tilings, we describe the arrangements of polyhedra. Our theory allows us to characterize polyhedral tilings, and thereby paves the way to study from short- to long-range order of disordered structures in a systematic way.

Disordered structures are often represented as polyhedral tilings^1^,^2^,^3^,^4^,^5^,^6^,^7^,^8^,^9^,^10^,^11^. For example, in studying the atomic structures of amorphous materials, the space can be divided into the so-called Voronoi polyhedra, where each polyhedron encloses its associated atom^1^,^2^,^3^,^4^,^5^,^6^,^7^,^8^. The shape of the polyhedron relates to how the associated atom is surrounded by its neighbouring atoms. The short-range order of a disordered structure can be thus characterized by what polyhedra constitute its associated polyhedral tiling, while the long-range order by how the polyhedra are arranged. So far, some attempts have been made to classify individual polyhedra^1^,^11^,^12^,^13^,^14^,^15^,^16^ and periodic tilings for crystals^17^,^18^. However, there have been no methods to describe what polyhedra are tiled in what way. If we could describe the arrangements of polyhedra, our understanding of the disordered structures would be deepened.

In this work, we create the theory of how the polyhedra are tiled. For this purpose, we use the hierarchy of structures of polytopes^19^,^20^: a polyhedron (3-polytope) is a tiling by polygons (2-polytopes), a polychoron (4-polytope) is a tiling by polyhedra, and so on. We first formulate a code for polyhedra (*p*_3_-code), and then generalize it to polychora. The code for polychora (*p*_4_-code) allows us to describe what polyhedra are tiled in what way.

## Results

### Polygon-sequence codeword

A polyhedron can be regarded as a tiling by polygons of the surface of a three-dimensional object that is topologically the same as a three-dimensional sphere. According to the idea developed by L. Euler, A. M. Legendre, F. Möbius, and P. R. Cromwell^19^, we assume that polygons are glued such that (1) any pair of polygons meet only at their sides or corners and that (2) each side of each polygon meets exactly one other polygon along an edge. In this picture, the vertex is a point on the polyhedron at which the corners of polygons meet (Supplementary Fig. S1a), and we say that the corners *contribute* to the vertex. We also say that a polygon (side) contributes to a vertex if one of its corners (endpoints) contributes to the vertex. Similarly, the edge is a line segment on the polyhedron along which the sides of polygons meet (Supplementary Fig. S1b). The interior area of a polygon is the face of the polyhedron.

We first deal with simple polyhedra, where every vertex is degree three. Here, the degree of a vertex is the number of edges connected to that vertex^21^. Afterwards, the method will be generalized to non-simple ones. We formulate the *p*_3_-code in such a way that the codeword of a polyhedron (simply, *p*_3_) instructs how to construct it from its building-block polygons. For this purpose, we combine a *polygon-sequence codeword* (*ps*_2_) and a *side-pairing codeword* (*sp*) as *p*_3_ = *ps*_2_;*sp*, where “;” is a separator.

The *ps*_2_-codeword is denoted as


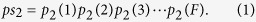


**Figure 1:**
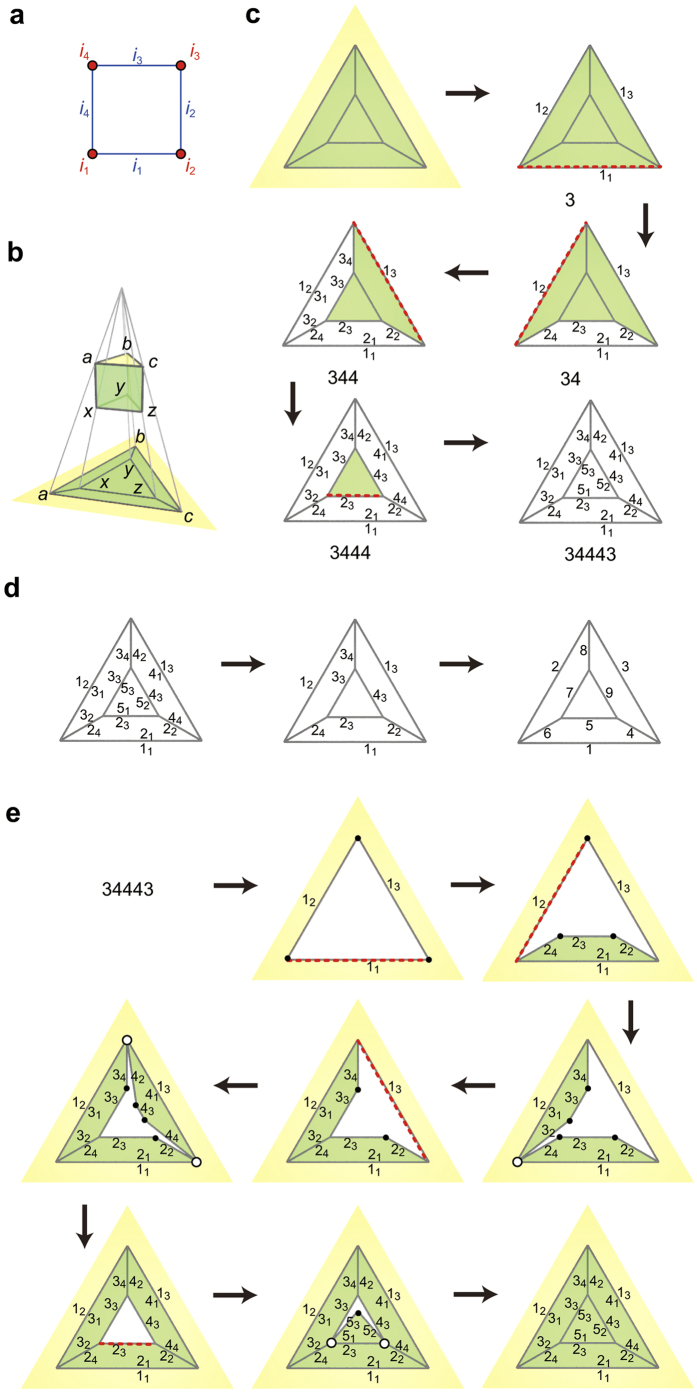
Encoding and decoding a polyhedron. Schlegel diagrams are used to illustrate polyhedra^20^,^22^. POV-Ray software^23^ is used to generate pictures. (**a**) Corner IDs (red) and side IDs (blue). (**b**) A Schlegel diagram is a projection of a polyhedron onto a plane. Note that the interior of the face *abc* on the polyhedron is mapped to the exterior of the outside face *abc* on the diagram. A counter CW direction around an inside polygon on the diagram, for example *z→x→a→c*, corresponds to a CW direction around its corresponding polygon on the polyhedron. (**c**) Encoding procedures. The red lines indicate the s-sides. (**d**) How to assign edge IDs. (**e**) Decoding procedures. Open circles indicate i-vertices, while filled circles indicate degree-one vertices.

Here, *p*_2_(*i*) is the number of sides on the polygon *i,* where *i* is the identification number (ID). *F* is the number of faces on the polyhedron. Generating *ps*_2_ thus reduces to assigning polygon IDs. To visually distinguish already-encoded polygons from to-be-encoded ones, we assume that all polygons are coloured at first, and make each polygon transparent when encoded. We call a side of a transparent polygon glued to a coloured one a *dangling side*. To identify each side, we introduce the side-ID *i*_*j*_. Here, the side *i*_*j*_ means the *j*th side of the polygon *i*, and the side-ID *i*_*j*_ represents an integer: *i*_*j*_ = *j* + 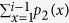. We also assign corner IDs so that the endpoints of the side *i*_*j*_ are the corners *i*_*j* + 1_ and *i*_*j*_ for 1 ≤ *j* < *p*_2_(*i*) (Fig. 1a). The smallest-ID dangling side (*s-side*) plays a key role in encoding. The *ps*_2_-codeword is generated as follows (Fig. 1b,c):

  - (a) Choose a side as the initial side, and the polygon 1 is the one having that side.
  - (b) Assign IDs (1_1_, 1_2_, 1_3_, ···, 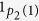) to the sides of the polygon 1 from the initial side in a clockwise (CW) direction.
  - (c) Make the polygon 1 transparent except for the corners and sides.
  - (a) The next polygon *i* (2 ≤ *i* ≤ *F*) is the coloured one glued to the s-side.
  - (b) Assign IDs (*i*_1_, *i*_2_, *i*_3_, ···, 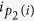) to the sides of the polygon *i* from the side glued to the s-side in a CW direction.
  - (c) Make the polygon *i* transparent except for the corners and sides.
  - (a) Repeat the procedure 2 until all polygons get transparent.

For latter convenience in formulating the *p*_4_-code, we assign edge IDs as follows. First we tentatively assign the smaller side ID to the edge, and then relabel the IDs so that the edge *i* is the one with the *i*th smallest tentative ID as illustrated in Fig. 1d. We also assign vertex IDs in a similar manner. First we tentatively assign the smallest corner ID to the vertex, and then relabel the IDs. Since the properties of simple polyhedra are not assumed, the algorithm for generating *ps*_2_ can be used to assign face, edge and vertex IDs not only to simple polyhedra, but also to non-simple ones.

We note that *p*_2_(*F* − 1) and *p*_2_(*F*) can be deduced from *p*_2_(1) *p*_2_(2) *p*_2_(3) ··· *p*_2_(*F* −2) (see Supplementary Note and Supplementary Fig. S2). However, we purposely admit the small redundancy in *ps*_2_ to explicitly express all information about the polygons of the polyhedron.

### Tentative side-pairing codeword

To formulate *sp* and the decoding algorithm, we first introduce the *zeroth tentative side-pairing codeword* (*tsp*^(0)^). We then formulate an algorithm to recover the original polyhedron from *ps*_2_*;tsp*^(0)^. Finally, we remove redundancy in *tsp*^(0)^ step-by-step to obtain *sp*.

**Figure 2:**
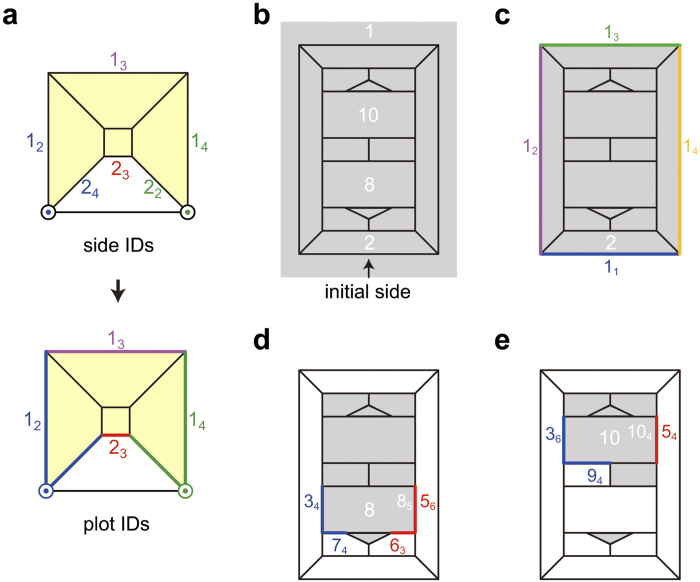
Illustration of plots, a-plots, and a-pairs. (**a**) How to form plots from dangling sides. The circled dotes are vertices contributed by two transparent polygons. (**b**) Polygon IDs. The polyhedron is encoded by choosing the side indicated by the arrow as the initial one, (**c**) *P*_1_. (**d**) *P*_7_. (**e**) *P*_9_.

We first introduce a *plot* as follows. A plot consists of a single dangling side or a chain of dangling sides. Here, two dangling sides are considered to be *chained* when they contribute to the same vertex contributed by two transparent polygons. Let *x* be the smallest side ID of a plot. We define the ID of that plot as *x* (Fig. 2a). We call the smallest-ID plot the *s-plot*. Note that all the sides of each plot are glued to the same coloured polygon.

If we encode a polyhedron twice, we know all the IDs of the polygons and sides from the beginning in the second time of encoding. When the polygon (*i* − 1) gets transparent, the coloured polygon *i* is glued to the s-side, so that the s-plot is glued to the polygon *i*. For example, in encoding the polyhedron shown in Fig. 2b, the polygon 2 in *P*_1_ is glued to the s-plot 1_1_ (Fig. 2c). Here, *P*_*i*_ is the object obtained when the polygon *i* gets transparent. In *P*_7_ (Fig. 2d), the polygon 8 is glued to the plot 5_6_ in addition to the s-plot 3_4_. We call such an additional plot an *a-plot*. The smallest-ID side of the a-plot 5_6_, which is the side 5_6_, is glued to the side 8_5_. We call such a pair an *a-pair* 8_5_5_6_. The sides 10_4_ and 5_4_ also form the a-pair 10_4_5_4_ (Fig. 2e).

By collecting the a-pairs, we define *tsp*^(0)^ as


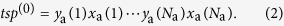


Here, the sides *y*_a_(*i*) and *x*_a_(*i*) form the a-pair *y*_a_(*i*)*x*_a_(*i*), where *y*_a_(*i*) > *x*_a_(*i*), and *y*_a_(*i*) < *y*_a_(*i* + 1). *N*_a_ is the number of a-pairs. For example, *tsp*^(0)^ of the polyhedron shown in Fig. 2b is 8_5_5_6_10_4_5_4_.

### Decoding algorithm

**Figure 3:**
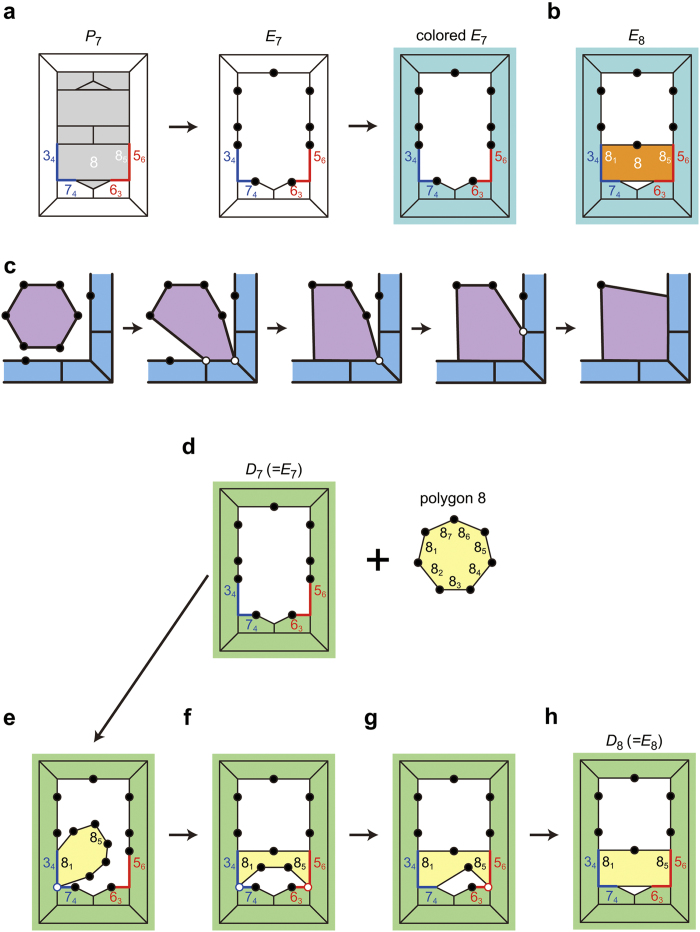
Procedures to recover a polyhedron. *D*_8_ (=*E*_8_) of the polyhedron shown in Fig. 2b can be constructed from *D*_7_ (=*E*_7_) and *ps*_2_;*tsp*^(0)^ = 458585574755433;8_5_5_6_10_4_5_4_. (**a**) How to construct *E*_7_ from *P*_7_. If we ignore colour, the coloured *E*_7_ is considered to be identical with *E*_7_. (**b**) *E*_8_ consists of the polygon 8 and *E*_7_. (**c**) How to rectify i-vertices (open circles). (**d**) Since *p*_2_(8) = 7, the polygon 8 is a 7-gon. (**e**) The side 8_1_ of the 7-gon is glued to the s-side 3_4_ of *D*_7_. An i-vertex is generated (blue circle). (**f**) We glue together the sides 8_5_ and 5_6_ as *tsp*^(0)^ instructs. Another i-vertex is generated (red circle). (**g**) We glue the sides 8_2_ and 7_4_ together to rectify the blue i-vertex. (**h**) We also glue the sides 8_4_ and 6_3_ together to rectify the red i-vertex. *D*_8_ thus obtained is identical with *E*_8_.

To formulate a decoding algorithm, we consider the sequence *D*_1_ *D*_2_ *D*_3_ ··· *D*_*F*_, where *D*_*i*_ is the partial polyhedron obtained when the polygon *i* is decoded. For the encoding process, we also consider the sequence *E*_1_ *E*_2_ *E*_3_ ··· *E*_*F*_. Here, *E*_*i*_ is the partial polyhedron obtained by removing the coloured polygons from *P*_*i*_ (Fig. 3a,b). For *D*_*i*_ and *E*_*i*_, if a side is not glued to the other polygon, we call it a dangling side. To define the plot, we consider a pair of two dangling sides to be chained if they contribute to the same vertex contributed by two polygons.

We formulate an algorithm to recover the original polyhedron from *ps*_2_;*tsp*^(0)^ so as to satisfy *D*_*i*_ = *E*_*i*_ at any *i*. If we assign side IDs (1_1_, 1_2_, 1_3_, ···, 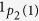) to a *p*_2_(1)-gon, then the resultant object *D*_1_ is identical with *E*_1_. Assume *D*_*i* − 1_ = *E*_*i* − 1_ for 2 ≤ *i* ≤ *F*. To construct *D*_*i*_ (=*E*_*i*_) from *D*_*i* − 1_ and *ps*_2_;*tsp*^(0)^, we introduce a rectification mechanism as follows. Since *D*_*i*_ is the partial polyhedron of a simple polyhedron, *D*_*i*_ must not have any degree-four vertex contributed by three polygons. We call such a vertex an *illegal vertex* (*i-vertex*). However, we allow intermediate products to transiently have i-vertices. When an i-vertex is generated, we rectify it by gluing together the two dangling sides contributing to it (Fig. 3c). Using the rectification mechanism, *D*_*i*_ can be constructed as follows. We first glue the side *i*_1_ of the polygon *i* to the s-side of *D*_*i* − 1_ (Fig. 3d,e). This is because *E*_*i*_ consists of the polygon *i* and *E*_*i* − 1_ in such a way that the side *i*_1_ of the polygon *i* is glued to the s-side of *E*_*i* − 1_. In addition, if *y*_a_(*n*) (1 ≤ *n* ≤ *N*_a_) is the side ID of the polygon *i*, then we glue the side *y*_a_(*n*) to the side *x*_a_(*n*) of *D*_*i* − 1_ (Fig. 3f) –for the polygon *i* in *E*_*i*_ is glued to not only the s-plot, but also the a-plot *x*_a_(*n*) of *E*_*i* − 1_ (Fig. 3b). If the product has i-vertices, we rectify them so that all the sides of the s-plot and a-plots get glued to the polygon *i* properly to satisfy *D*_*i*_ = *E*_*i*_ (Fig. 3g,h).

To summarize, the original polyhedron can be recovered from *ps*_2_;*tsp*^(0)^ as follows (Fig. 1e):

  - (a) The polygon 1 is a *p*_2_(1)-gon.
  - (b) Assign IDs (1_1_, 1_2_, 1_3_, ···, 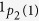) to its sides in a CW direction. The resultant object is *D*_1_.
  - (a) The next polygon *i* (2 ≤ *i* ≤ *F*) is a *p*_2_(*i*)-gon.
  - (b) Assign IDs (*i*_1_, *i*_2_, *i*_3_, ···, 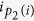) to its sides in a CW direction.
  - (c) Glue the side *i*_1_ of the polygon *i* to the s-side of *D*_*i* − 1_.
  - (d) If *y*_a_(*n*) (1 ≤ *n* ≤ *N*_a_) is the side ID of the polygon *i*, then glue the side *y*_a_(*n*) to the side *x*_a_(*n*) of *D*_*i* − 1_.
  - (e) If i-vertices are generated, then rectify them, and repeat this procedure until no i-vertices remain. The resultant object is *D*_*i*_.
  - (a) Repeat the procedure 2 until all polygons are placed.

### Side-pairing codeword

In decoding *ps*_2_;*tsp*^(0)^, *D*_*i*_ = *E*_*i*_ at any *i*. However, what we need is just *D*_*F*_ = *E*_*F*_. By allowing *D*_*i*_ ≠ *E*_*i*_ for 1 ≤ *i* ≤ *F* − 1, we can make the more compact *sp* as described below.

We remove redundancy in *tsp*^(0)^ step-by-step. To examine *y*_a_(*N*_a_)*x*_a_(*N*_a_) for necessity, we consider the *first test codeword* denoted as


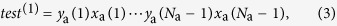


which is obtained by stripping *y*_a_(*N*_a_)*x*_a_(*N*_a_) off from *tsp*^(0)^. Then we attempt to decode *ps*_2_;*test*^(1)^. When the polygon having the side *y*_a_(*N*_a_) is decoded, we will fail to glue the sides *y*_a_(*N*_a_) and *x*_a_(*N*_a_) together, because the a-pair *y*_a_(*N*_a_)*x*_a_(*N*_a_) is missing in *test*^(1)^. However, we can proceed decoding. If the missing a-pair is cured and the original polyhedron is successfully reproduced in the subsequent decoding process, then we call the a-pair the *curable a-pair*. Otherwise, we call it the *non-curable a-pair*. If the a-pair is curable, then we can remove *y*_a_(*N*_a_)*x*_a_(*N*_a_) from *tsp*^(0)^. We therefore set the first tentative side-pairing codeword as *tsp*^(1)^ = *test*^(1)^. On the other hand, if the a-pair is non-curable, then *tsp*^(1)^ = *tsp*^(0)^.

To examine *y*_a_(*N*_a_+1 − *i*)*x*_a_(*N*_a _+ 1 − *i*) (2 ≤ *i* ≤ *N*_a_) for curability, we consider the *i*th test codeword *test*^(*i*)^, which is obtained by stripping *y*_a_(*N*_a_ + 1 − *i*)*x*_a_(*N*_a_ + 1 − *i*) off from *tsp*^(*i − *1)^. Then we attempt to decode *ps*_2_;*test*^(*i*)^. If the a-pair is curable, then *tsp*^(*i*)^ = *test*^(*i*)^, otherwise, *tsp*^(*i*)^ = *tsp*^(*i − *1)^.

We repeat the above-mentioned procedure, and 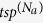 is what we call *sp*. For example, *tsp*^(0)^ of the polyhedron shown in Fig. 2b is 8_5_5_6_10_4_5_4_. The a-pair 10_4_5_4_ is non-curable (Supplementary Fig. S3a), while the a-pair 8_5_5_6_ is curable (Supplementary Fig. S3b). As a result, *sp* = 10_4_5_4_. Since the polyhedron is encoded as 458585574755433: 10_4_5_4_, we call it a 458585574755433;10_4_5_4_-polyhedron, or a 4(58)^2^5^2^7475^2^43^2^;10_4_5_4_-polyhedron for short.

### Representative codeword

Our encoding starts with choosing an initial side. A different initial side for the same polyhedron may give a different *p*_3_-codeword. There are 2*E* possible initial sides for a polyhedron. Here, *E* is the number of edges on the polyhedron. We also examine the mirror-image polyhedron, which gives additional 2*E* possibilities. A maximum of 4*E* different codewords can be obtained from a polyhedron and its mirror image. To determine the representative one, we introduce the lexicographical number Lex(*p*_3_). Given that *ps*_2_ and *sp* can be read as positive *F*- and 2*N*_na_-digit integers, we define Lex(*p*_3_) as the concatenation of the two numbers. Here, *N*_na_ is the number of non-curable a-pairs. We use the codeword with the smallest Lex(*p*_3_) as the representative one.

### Non-simple polyhedron

On the analogy of the *n*-regular graph^21^, we call the polyhedron whose vertices are all degree *n* the *n*-regular polyhedron. 3-regular polyhedra are simple, while, for *n* > 3, *n*-regular polyhedra are non-simple. In encoding [decoding], if we regard that two dangling sides are chained when they contribute to the same vertex contributed by (*n *− 1) transparent polygons [polygons] and modify an i-vertex to be a vertex contributed by a pair of two dangling sides and *n* transparent polygons [polygons], our *p*_3_-code is straightforwardly applicable to *n*-regular polyhedra. For example, the octahedron is encoded as “4-regular 3^8^”. The icosahedron is “5-regular 3^20^”. However, if a non-simple polyhedron is non-regular, this method cannot be used. To deal with all non-simple polyhedra, we formulate a method that uses a one-to-one correspondence between a non-simple polyhedron and its associated simple one as described below.

**Figure 4:**
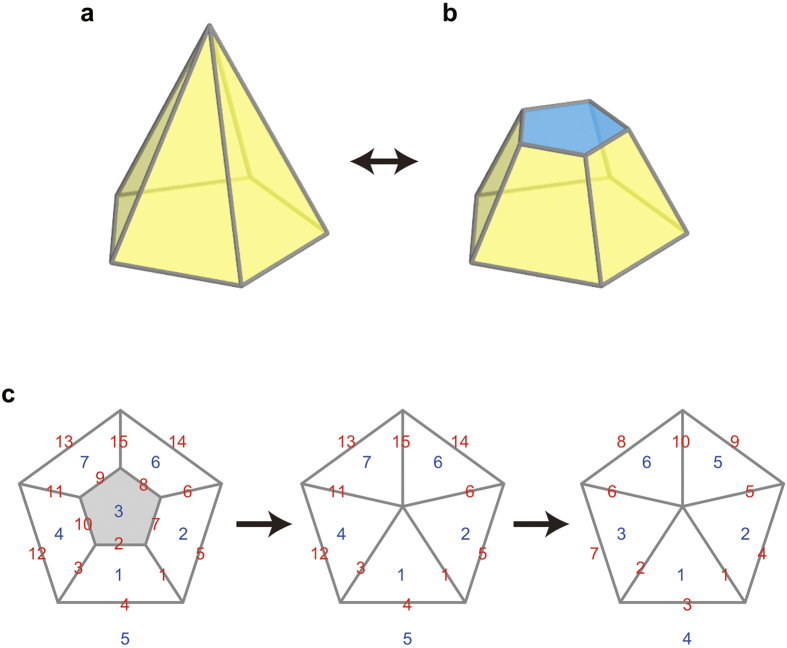
Non-simple polyhedron. (**a**) A pentagonal pyramid. (**b**) The simple polyhedron obtained by cutting the degree-five vertex of the pentagonal pyramid shown in (**a**). The cross section is coloured blue. (**c**) How to assign edge and face IDs, being expressed with Schlegel diagrams. We first assign edge and face IDs to the associated simple polyhedron. If we shrink the cross sections to the vertices, some edges and faces disappear. We therefore relabel the edge and face IDs. The red and blue numbers are edge and face IDs, respectively.

Any non-simple polyhedron can be transformed into its associated simple polyhedron by cutting every vertex of degree *d* (>3) and replacing it with a *d*-gonal cross section^22^. For example, a non-simple pentagonal pyramid can be transformed into a pentagonal prism by cutting the apex (Fig. 4a,b). By marking the cross sections, a one-to-one correspondence can be established between any non-simple polyhedron and its associated simple one. Using the one-to-one correspondence, we encode a non-simple polyhedron. Here, we modify the s-side to be the smallest-ID dangling side in real polygons (not cross sections) so that the face, edge, and vertex IDs of a non-simple polyhedron determined from its associated simple polyhedron using an algorithm described below conform to those determined directly from itself using the algorithm described above. The *p*_3_-codeword of a non-simple polyhedron is generated as follows:

1. Choose a side of a non-simple polyhedron as an initial side.
2. Construct its associated simple polyhedron by cutting every vertex of degree *d* (>3) and replacing it with a *d*-gonal cross section.
3. Encode the associated simple polyhedron from the initial side corresponding to the one determined in the procedure 1.
4. Put a dot on *p*_2_(*i*) if the polygon *i* is the cross section; for example, a codeword for the pentagonal pyramid is 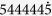, or 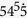 for short, indicating that the polygon 7 is a cross section.
5. The edge and face IDs can be assigned as illustrated in Fig. 4c.

Since *ps*_2_ contains dots, it is not a number. We therefore define Lex(*ps*_2_) as the concatenation of two numbers *ps*_2_^(1)^ and *ps*_2_^(2)^. Here, *ps*_2_^(1)^ is a number obtained from *ps*_2_ by replacing every number without a dot to 0 and then by removing all dots, while *ps*_2_^(2)^ is obtained by removing all dots from *ps*_2_. For example, Lex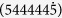 is the concatenation of 0000005 and 5444445, namely, 00000055444445.

Note that, if a vertex is concave, cutting the vertex may not be well defined. However, by assuming that a polyhedron is flexible, we can inflate it so that a concave vertex becomes a convex one. Then we can cut the vertex.

Decoding is achieved easily. We first construct the associated simple polyhedron, and then shrink the cutting sections to the vertices.

The duality of polyhedra can be used to make a codeword more compact. Every polyhedron has its associated dual, which is constructed as follows^21^:

1. Draw a vertex *v*_i_^*^ of the dual polyhedron on each face *f*_*i*_ of the original polyhedron.
2. Draw an edge *e*_ij_^*^ of the dual, if the faces *f*_*i*_ and *f*_*j*_ share an edge *e*, to connect the vertices *v*_i_^*^ and *v*_j_^*^ such that *e*_ij_^*^ crosses *e*, but does not cross the other edges.

**Figure 5:**
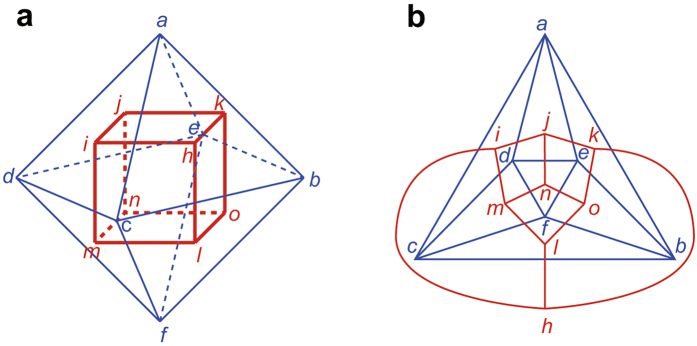
Duality. (**a**) Dual of an octahedron (blue) is a hexahedron (red). (**b**) Duality illustrated in (**a**) is expressed with Schlegel diagrams.

There is a one-to-one correspondence between the original polyhedron and its dual. For example, the dual of an octahedron is a hexahedron (Fig. 5a,b). Reversely, the dual of a hexahedron is an octahedron (Fig. 5b). A *p*_3_-codeword for the octahedron is 66(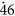)^6^, while a *p*_3_-codeword for the hexahedron is 4^6^. Using the duality, we encode the octahedron as ^★^4^6^. Here, “^★^” indicates that the octahedron is the dual of the 4^6^-polyhedron. We define Lex (^★^4^6^) as the concatenation of Lex (^★^), which we define as 1, and Lex(4^6^). Therefore, Lex (^★^4^6^) is 1444444. On the other hand, Lex(66(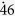)^6^) is 0040404040404066464646464646. Since Lex (^★^4^6^) is the smallest, the representative *p*_3_ for the octahedron is ^★^4^6^, which is more compact than 66(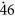)^6^. We also note that the hexahedron can be encoded as ^★^66(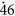)^6^, but it is not the representative codeword. For reference, the representative *p*_3_-codewords for the tetrahedron, hexahedron, octahedron, dodecahedron, and icosahedron are 3^4^, 4^6^, ^★^4^6^, 5^12^, and ^★^5^12^, respectively.

Consider that we calculate *p*_3_ for a polyhedron by encoding its dual for a given initial side of the original polyhedron. To determine the initial side for encoding the dual, we use the one-to-one correspondence between the original polyhedron and its dual. For example, in encoding an octahedron shown in Fig. 5, when we choose the side *cb* of the polygon *cbf* as the initial side, the edge *cb* is the edge 1 and the vertex *c* is the vertex 1. The edge and vertex are mapped to the edge *hl* and face *hlmi* of the dual. Using this relation, we choose the initial side for encoding the dual so that 1s are assigned to the edge *hl* and face *hlmi*. Thus, the initial side is determined to be the side *hl* of the polygon *hlmi*.

Our *p*_3_-code is more robust and efficient than the previous methods for polyhedra^1^,^11^,^12^,^13^,^14^,^15^,^16^ (see Supplementary Note). However, what is really stupendous is that only our theory can be generalized to polyhedral tilings as described below.

### Code for polychora

We regard a polychoron as a tiling by polyhedra of the surface of a four-dimensional object that is topologically the same as a four-dimensional sphere. We assume that polyhedra are glued together such that (1) any pair of polyhedra meet only at their faces, edges, or vertices and that (2) each face of each polyhedron meets exactly one other polyhedron along a ridge. The 0-face, peak, and ridge are a point, line segment, and area on the polychoron, where the vertices, edges, and faces of polyhedra meet, respectively (Supplementary Fig. S1c–e). The interior space of a polyhedron is the cell of the polychoron.

We first deal with polychora whose peaks are all contributed by three polyhedra. Afterwards, the method will be generalized to polychora in general. The *p*_4_-codeword consists of a *polyhedron-sequence* codeword (*ps*_3_) and a *face-pairing* codeword (*fp*), and is denoted as *p*_4_ = *ps*_3_;*fp*.

**Figure 6:**
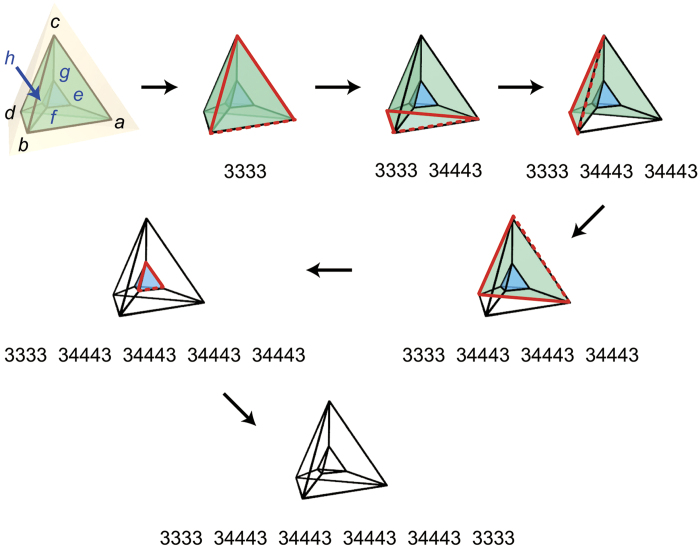
Encoding a polychoron. Procedures are illustrated with three-dimensional Schlegel diagrams (a projection from four- to three-dimensional space). The polychoron *abcdefgh* consists of two 3333-polyhedra and four 34443-polyhedra. The face *abc* and edge *ab* of the outside polyhedron *abcd* are chosen as the initial face and edge, respectively. The red lines indicate the s-face, and the dashed one indicates the smallest-ID edge of the s-face. See Supplementary Fig. S4 for details in the edge and face IDs. Note that a counter CW direction around a face of inside polyhedra on the Schlegel diagram, for example *f→e→g* around the face *feg* of the polyhedron *fegh*, corresponds to a CW direction around its corresponding face on the polychoron in four-dimensional space.

The *ps*_3_-codeword is denoted as


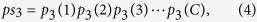


where *C* is the number of cells on the polychoron, and *p*_3_(*i*) is the *p*_3_-codeword for the polyhedron *i*. To assign IDs to the polyhedra, we use edge (face) IDs *i*_*j*_. Here, the edge (face) *i*_*j*_ is the *j*th edge (face) of the polyhedron *i*. In encoding, each polyhedron is coloured at first, but gets transparent when encoded. We call a face of a transparent polyhedron glued to a coloured one a *dangling face*. The smallest-ID dangling face (*s-face*) plays a key role in encoding. The *ps*_3_-codeword is generated as follows (Fig. 6 and Supplementary Fig. S4):

  - (a) Choose a face of a polyhedron and an edge of that face as the initial face and edge, respectively; the polyhedron 1 is the one having the initial face.
  - (b) Determine *p*_3_(1) by encoding the polyhedron 1 in such a way that the face 1_1_ (edge 1_1_) becomes the initial face (edge).
  - (c) Make the polyhedron 1 transparent except for the vertices and edges.
  - (a) The next polyhedron *i* (2 ≤ *i* ≤ *C*) is the coloured one glued to the s-face.
  - (b) Determine *p*_3_(*i*) by encoding the polyhedron *i* in such a way that the face *i*_1_ (edge *i*_1_) is glued to the s-face (the smallest-ID edge of the s-face).
  - (c) Make the polyhedron *i* transparent except for the vertices and edges.
  - (a) Repeat the procedure 2 until all polyhedra get transparent.

As with the case of polyhedra, this method can be used to assign cell, ridge, peak, and 0-face IDs to polychora in general.

To define *zeroth tentative face-pairing codeword tfp*^(0)^, we explain plots for polychora. If a pair of two dangling faces contribute to a peak contributed by two transparent polyhedra, the dangling faces are considered to be *chained*. A single dangling face or chained dangling faces form a plot; the plot here is a two-dimensional object. We assign plot IDs so that the smallest-ID face of the plot *x* is the face *x*. If the polyhedron *i* in *P*_*i* − 1_ is glued to plots other than the s-plot, we call them *a-plots*. By the a-pair *wzv*, we mean that the face *w* (of the polyhedron *i*) is glued to the face *v* of the a-plot *v* in such a way that the edge *z* (of the face *w*) is glued to the smallest-ID edge of the face *v.* By collecting the a-pairs, *tfp*^(0)^ is denoted as


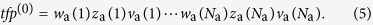


Here, *w*_a_(*i*) > *v*_a_(*i*) and *w*_a_(*i*) < *w*_a_(*i* + 1).

In decoding, by a *dangling face*, we mean a face that is not glued to the other polyhedron. If a pair of dangling faces contribute to a peak that is also contributed by three polyhedra, we call that peak an *illegal peak* (*i-peak*). The i-peak can be rectified by gluing together the two dangling faces contributing to it. The original polychoron can be recovered from its *ps*_3_;*tfp*^(0)^ as follows (Supplementary Figs S5 to S7):

1.

- (a) Decode *p*_3_(1) to obtain the polyhedron 1, assigning face and edge IDs.

2.

- (a) Decode *p*_3_(*i*) to obtain the next polyhedron *i* (2 ≤ *i* ≤ *C*), assigning face and edge IDs.
- (b) Glue the face *i*_1_ of the polyhedron *i* to the s-face of *D*_*i*−1_ in such a way that the edge *i*_1_ is glued to the smallest-ID edge of the s-face.
- (c) If *w*_a_(*n*) (1 ≤ *n* ≤ *N*_a_) is the face ID of the polyhedron *i*, then glue the face *w*_a_(*n*) to the face *v*_a_(*n*) of *D*_*i*−1_ in such a way that the edge *z*_a_(*n*) is glued to the smallest-ID edge of the face *v*_a_(*n*).
- (d) If i-peaks are generated, then rectify them, and repeat this procedure until no i-peaks remain.

3.

- (a) Repeat the procedure 2 until all polyhedra are placed.

By an similar argument for *sp*, the redundancy in *tfp*^(0)^ can be removed step-by-step with generating *tfp*^(1)^, *tfp*^(2)^, *tfp*^(3)^, ···, and *tfp*^(*N*a)^ is what we call *fp*.

A maximum of 12*P* different *p*_4_-codewords can be obtained from a polyhedron and its mirror image, where *P* is the number of peaks on the polychoron. Given that *ps*_3_ and *fp* can be read as *C*- and 3*N*_na_ -digit numbers, respectively, we define Lex(*p*_4_) as the concatenation of the two numbers. We use the lexicographically smallest *p*_4_ as the representative one.

### Affected polychora

We first define the *degree of a peak* as the number of polyhedra contributing to that peak. We call a peak of degree more than three an *affected peak*. We say a polychoron without an affected peak to be *non-affected*. Reversely, an *affected polychoron* has one or more affected peaks. The *p*_4_-code for non-affected polychora formulated above can be generalized to affected ones by using a one-to-one correspondence between an affected polychoron and its associated non-affected one as described below.

Any affected polychora can be transformed into a non-affected one by cutting its affected peaks. However, when different affected peaks are incident to the same 0-face, different non-affected polychora are obtained depending on the order of cutting. Therefore, we first assign peak IDs, and then cut the affected peaks in the ascending order of peak ID.

**Figure 7:**
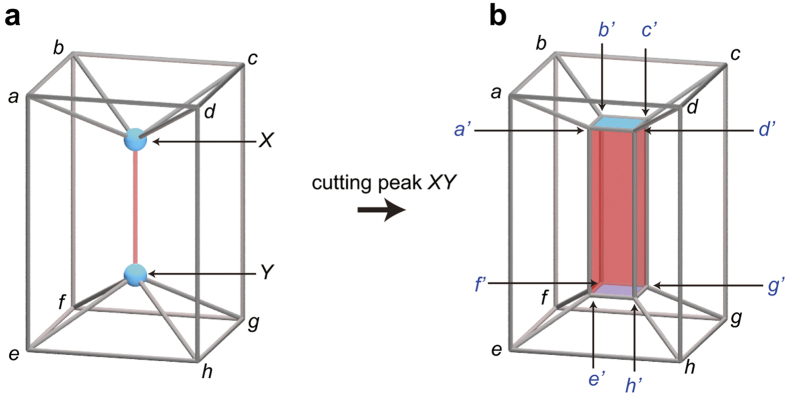
How to deal with affected peaks. (**a**) Three-dimensional Schlegel diagram of a polychoron *abcdefghXY*. The hexahedron *abcdefgh* is the outside polyhedron. The red peak *XY* is an affected peak contributed by four polyhedra *abfeXY, bcgfXY, dcghXY*, and *adheXY*. (**b**) Three-dimensional Schlegel diagram of a polychoron obtained by cutting the affected peak *XY*.

Suppose that we create a cross-section cell (cs-cell) by cutting an affected peak *XY*, connecting the 0-faces *X* and *Y*. We say its 0-face to be type-*X* (type-*Y*), if it is the cross section of a peak incident to *X* (*Y*). The ridges of the cs-cell are classified into three types: type-*X* ridges consisting of only type-*X* 0-faces, type-*XY* ridges consisting of both type-*X* and type-*Y* 0-faces, and type-*Y* ridges consisting of only type-*Y* 0-faces. In other words, by cutting a peak *XY*, it is mapped to a cs-cell in such a way that its endpoints are mapped to either type-*X* or type-*Y* ridges. Note that the type-*X* and -*Y* ridges do not adjoin each other because the type-*XY* ridges separate them, and that the number of the type-*XY* ridges is the same as the degree of the peak *XY*. In the example shown in Fig. 7, by cutting the affected peak *XY* of degree four, the 0-faces *X* and *Y* are mapped to the cross-section ridges *a′b′c′d′* and *e′f′g′h′*, respectively. Four cross-section ridges *a′d′h′e′, b′a′e′f′, c′b′f′g′*, and *d′c′g′h′* are type-*XY*.

Based on the above discussion, an affected polychoron can be encoded as follows:

1. Choose a face and an edge of an affected polychoron as an initial face and edge, and then assign peak IDs.
2. Cut the affected peaks in the ascending order of peak ID.
3. Encode the associated non-affected polychoron from the initial face and edge corresponding to the ones used in procedure (1).
4. To identify the cs-cell that is created when we cut an affected peak, we denote, for example, the *p*_3_-codeword for a cs-hexahedron as 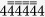. Here, four double lines on 4 designate that the cs-hexahedron is mapped from the affected peak of degree four and that the four 4-gonal faces contribute to the type-*XY* ridges.

For example, a *p*_4_-codeword for a polychoron shown in Fig. 7a is 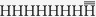, where H = 4^6^ and 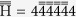.

Decoding is achieved easily. We first reproduce the associated non-affected polychoron, and then shrink the cs-cells to the corresponding affected peaks.

To define Lex(*p*_4_), we define Lex(*ps*_3_) for a non-simple polychoron as the concatenation of Lex(*ps*_3_^(1)^) and Lex(*ps*_3_^(2)^). Here, *ps*_3_^(1)^ is the codeword obtained from *ps*_3_ as follows. We first replace every *p*_3_ with 0 except *p*_3_s for the cs-cells corresponding to the affected peaks. We then deal with the *p*_3_s for the affected peaks, and replace the digits without double lines with 0 and remove the double lines. The *ps*_3_^(2)^-codeword is the one obtained by removing double lines from *ps*_3_. For example, Lex(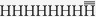) is the concatenation of 0000000X and HHHHHHHH, namely, 0000000XHHHHHHHH. Here, X = 044440.

As in the case of polyhedra, the duality of polychora^20^ can be used to make a codeword more compact. For example, by using the duality, the representative *p*_4_-codewords for 5-, 8-, 16-, 24-, 120-, and 600-cells^12^ are encoded as T^4^, H^8^, ^★^H^8^, O^24^, D^12^, and ^★^D^12^, respectively. Here, T, O, and D are the representative *p*_3_-codewords for the tetrahedron 3^4^, octahedron ^★^H, and dodecahedron 5^12^, respectively. Note that “^★^” in “^★^H^8^” indicates the dual of H^8^, and Lex (^★^*p*_4_) is defined as the concatenation of Lex (^★^) and Lex(*p*_4_).

### Describing a polyhedral tiling

**Figure 8:**
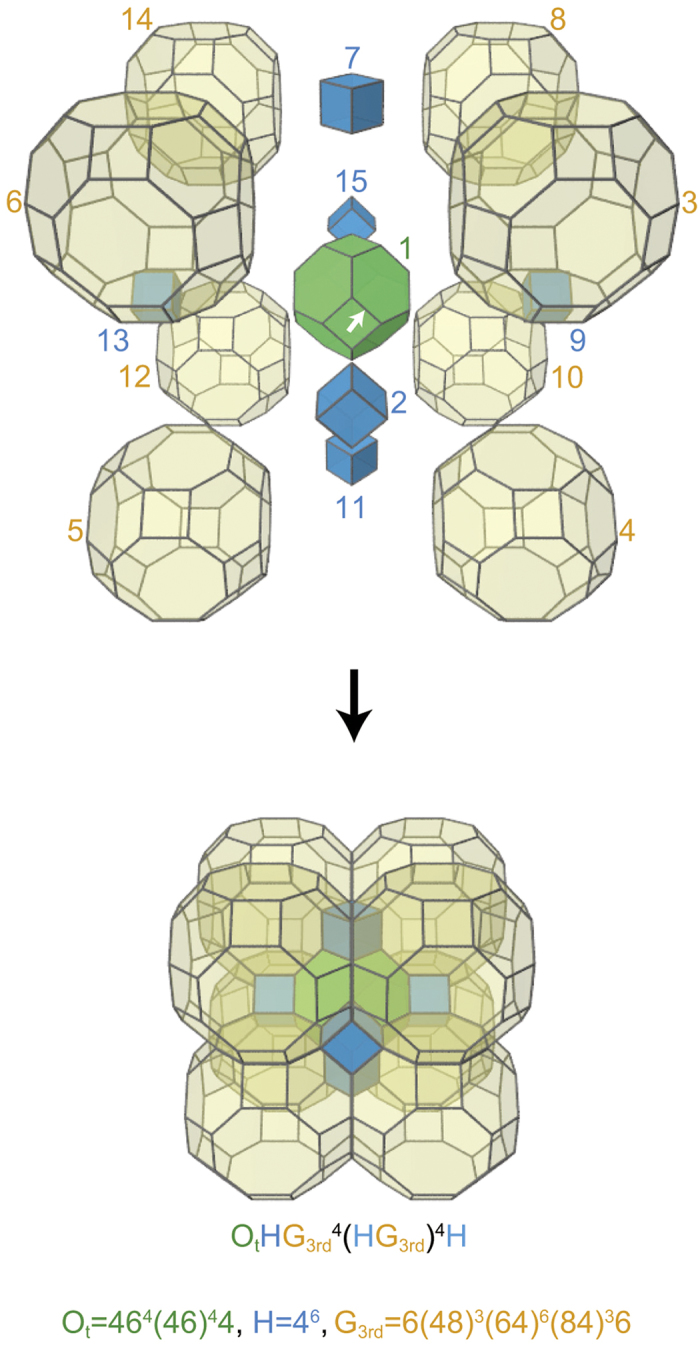
Codeword for a complex of polyhedra. A central truncated octahedron (green) is surrounded by six hexahedra (blue) and eight great rhombicuboctahedra (yellow). By choosing the 4-gon having the initial edge indicated by the arrow as the initial face, the arrangement is encoded as O_t_HG_3rd_^4^(HG_3rd_)^4^H. The numbers near the polyhedra are their polyhedron IDs.

Since a complex of polyhedra can be regarded as a partial polychoron, the *p*_4_-code can be used to describe the arrangement of polyhedra in polyhedral tilings. The complex of polyhedra shown in Fig. 8, for example, is encoded as a partial *p*_4_-codeword O_t_HG_3rd_^4^(HG_3rd_)^4^H. Here, by a partial *p*_4_, we mean that decoding it results in a partial polychoron. O_t_ (=46^4^(46)^4^4) and H (=4^6^) are the representative *p*_3_s of the truncated octahedron and hexahedron, respectively. G_3rd_ (=6(48)^3^(64)^6^(84)^3^6) is the third smallest *p*_3_ of the great rhombicuboctahedron.

## Discussion

In this work, we have created the theory of polyhedral tilings, which allows us to convert a local arrangement of polyhedra into a partial *p*_4_-codeword that represents what polyhedra are tiled in what way. Traditionally, the index *c*_3_*c*_4_*c*_5_∙∙∙ has been used to classify polyhedra in polyhedral tilings of liquids, glasses, grains and foams, where *c*_i_ is the number of i-gons on the polyhedron^1^,^2^,^3^,^4^,^5^,^6^,^9^,^11^. However, the index sometimes fails to distinguish polyhedra with different structures, which prevents a close investigation of disordered structures. For example, the 35664453-polyhedron and 34566543-polyhedrdon have the same index, 2222000···. Although the Weinberg code may be a possible remedy and has been used recently^11^, its codeword is lengthy and therefore redundant, still hampering our understanding of disordered structures. For example, the Weinberg codeword of the most frequently found polyhedron in the Poisson-Voronoi tessellation is “ABCACDEFAFGHIBIJKDKLELMGMNHNJNMLKJIHGFEDCB”. In contrast, using our *p*_3_ code, the same polyhedron is encoded as “356645445”. We also note that when the Voronoi polyhedron associated with an atom is encoded as 356645445, the atom is surrounded by neighbouring atoms forming a ★356645445-polyhedron. Our method thus allows us to describe polyhedral tilings succinctly, which is essential to understand disordered structures. In addition, although the short-range order can be studied by the previous methods, the long-range order cannot. Only our theory allows us to characterize what polyhedra are tiled in what way, and thereby paves the way to study from short- to long-range order of disordered structures in a systematic way. Moreover, our theory can be generalized to higher-dimensional polytopes to study disordered structures of any dimension.

## Additional Information

**How to cite this article**: Nishio, K. and Miyazaki, T. How to describe disordered structures. *Sci. Rep.* **6**, 23455; doi: 10.1038/srep23455 (2016).

## Supplementary Material

Supplementary Information
