# Supplementary material for: How to describe disordered structures
Source: Sci Rep. 2016 Apr 11;6:23455. doi: 10.1038/srep23455 (PMC4827397; doi:10.1038/srep23455)
Supplement: Supplementary Information [file srep23455-s1.pdf]

Supplemental Information for "How to describe disordered structures"

Kengo Nishio and Takehide Miyazaki

Correspondence should be addressed to K. N. (k-nishio@aist.go.jp).

Contents

Supplementary Figures S1 to S7

Supplementary Note

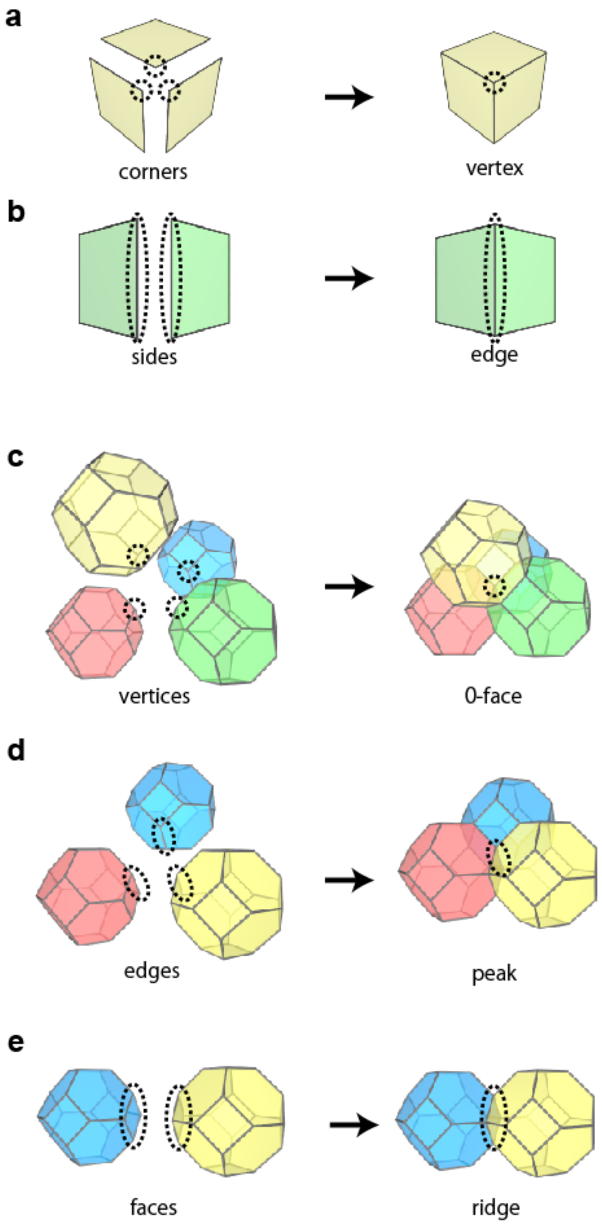


**Supplementary Figure S1 | Parts of a polyhedron and polychoron. a**, The vertex is a point on the polyhedron where the corners of polygons meet. **b**, The edge is a line segment on the polyhedron where the sides of polygons meet. **c**, The 0-face is a point on the polychoron where the vertices of polyhedra meet. **d**, The peak is a line segment on the polychoron where the edges of polyhedra meet. **e**, The ridge is an area on the polychoron where the faces of polyhedra meet.

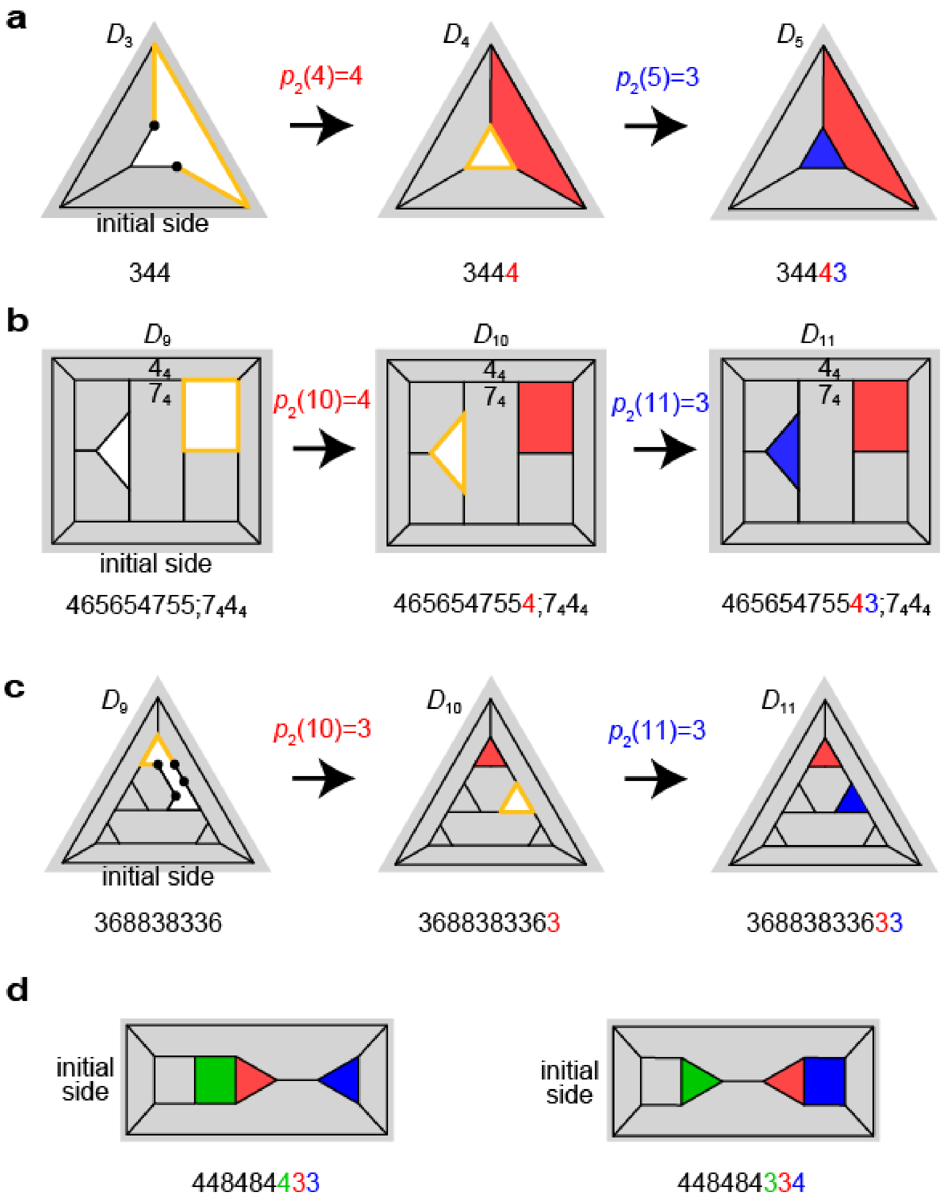


**Supplementary Figure S2 | How to determine $p_2(F - 1)$ $p_2(F)$ from $p_2(1)$ $p_2(2)$ $p_2(3)$ ··· $p_2(F - 2)$;*sp*. a**, 34443-polyhedron (Case 1). We can construct $D_3$ from 344. The s-plot coloured orange is composed of three sides, so that $L(3) = 3$. Since there remain only two plots adjoining each other, $p_2(4) = L(3) + 1 = 4$. We can then construct $D_4$ from 3444, from which we find $p_2(5) = L(5) = 3$. **b**, $46565475543;7_44_4$-polyhedron (Case 2I). Since there remain two separate plots in $D_9$, $p_2(10) = L(9) = 4$. From $D_{10}$, we find $p_2(11) = L(10) = 3$. **c**, 36883833633-polyhedron (Case 2II). Since there remain four plots in $D_9$, $p_2(10) = L(9) = 3$. From $D_{10}$ , we find $p_2(11) = L(10) = 3$. **d**, Different 448484433- and 448484334-polyhedra have the same $p_3^{(-3)}$ (= 448484), so that $p_2(F\text{-}2)$ cannot be determined from $p_3^{(-3)}$.

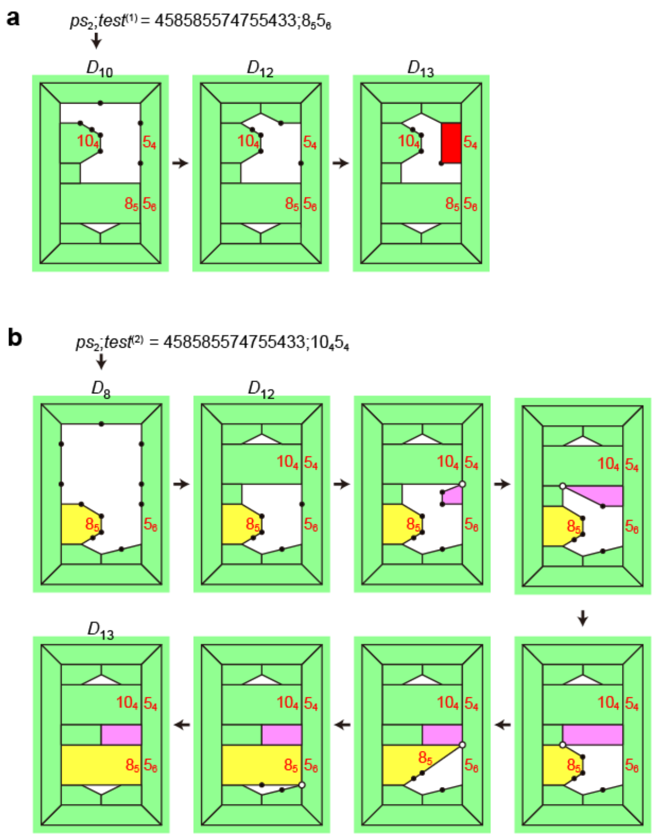


**Supplementary Figure S3 | How to generate *sp*.** The $ps_2;tsp^{(0)}$-codeword of the polyhedron shown in Fig. 2b is 458585574755433;$8_5 5_6 10_4 5_4$. **a**, By stripping off $10_4 5_4$ from $tsp^{(0)}$, $test^{(1)} = 8_5 5_6$. In decoding $ps_2;test^{(1)}$, the red 4-gon is incorrectly glued to the side $5_4$, so that the original polyhedron cannot be recovered. Since the a-pair $10_4 5_4$ turns out to be non-curable, $tsp^{(1)} = 8_5 5_6 10_4 5_4$. **b**, By stripping off $8_5 5_6$ from $tsp^{(1)}$, $test^{(2)} = 10_4 5_4$. In decoding $ps_2;test^{(2)}$, although we fail to glue the side $8_5$ to the side $5_6$ when the yellow polygon 8 is decoded ($D_8$), the missing a-pair is cured when the pink polygon 13 is decoded ($D_{13}$). Since the a-pair $8_5 5_6$ turns out to be curable, $sp = tsp^{(2)} = 10_4 5_4$.

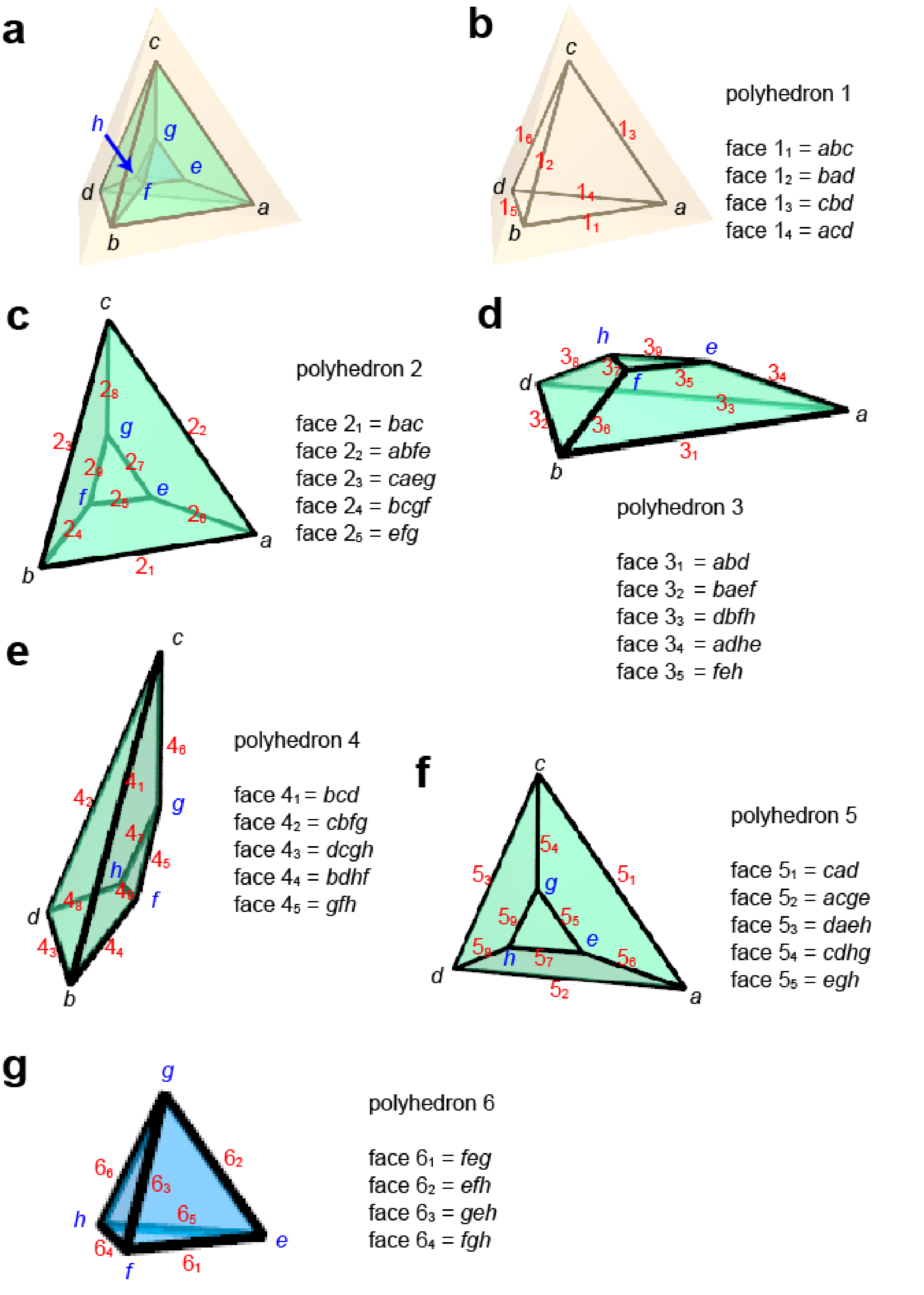


**Supplementary Figure S4 | Edge and face IDs of the polychoron *abcdefgh* shown in Fig. 6.**

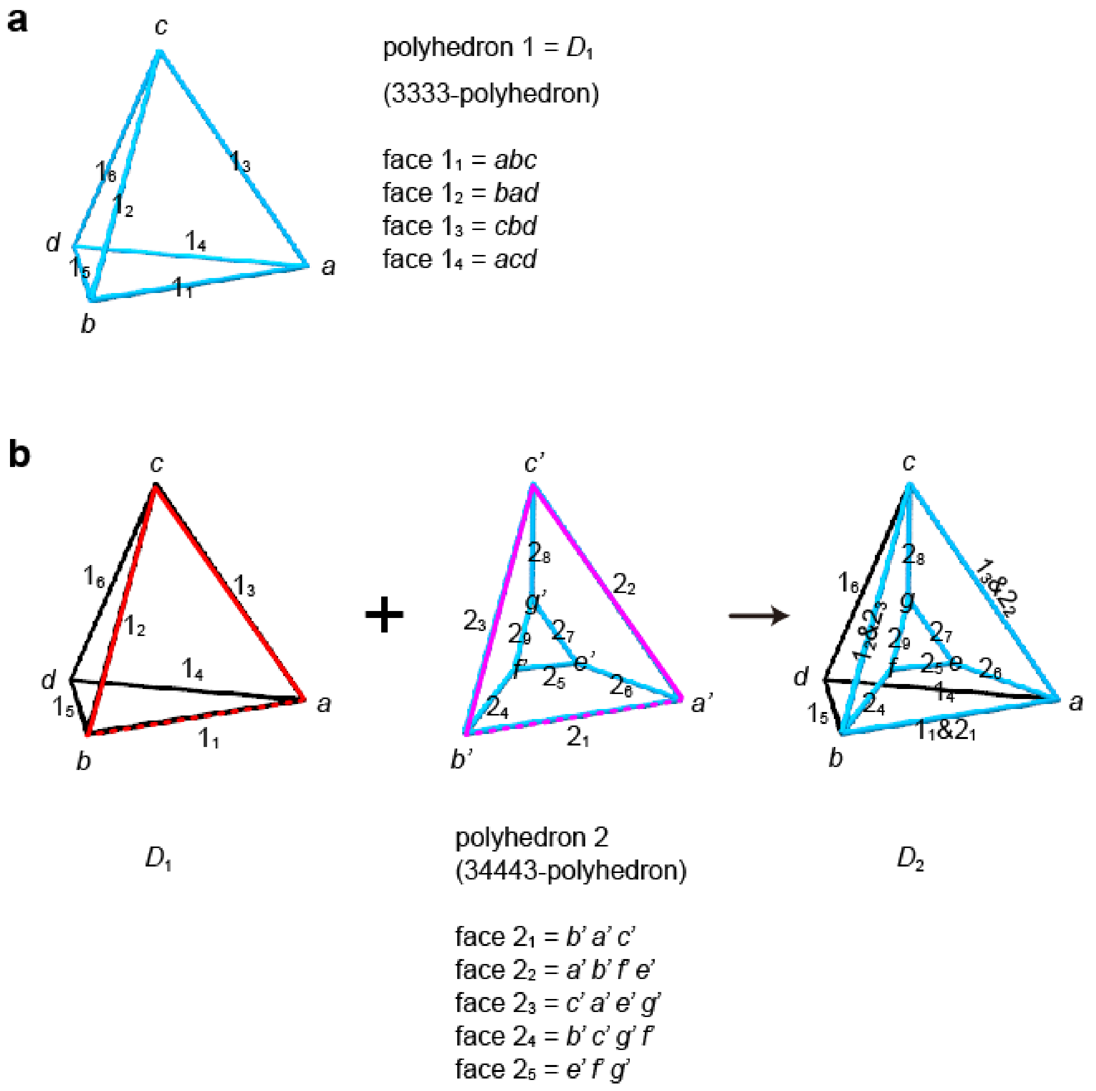


**Supplementary Figure S5 | How to decode 3333 34443 34443 34443 34443 3333 (Part 1).** Procedures are illustrated with three-dimensional Schlegel diagrams. The polyhedron 1 is assumed to be the outside polyhedron. **a**, The 3333-polyhedron *abcd* is the polyhedron 1. $D_1$ is the polyhedron 1 itself. The edge IDs $1_1$, $1_2$, $1_3$, ···, $1_6$ are shown near the corresponding edges. **b**, The polyhedron *b'a'c'f'e'g'* is the polyhedron 2. Since the polyhedron 2 is an inside polyhedron, a counter CW direction such as *b'→a'→c'* around the face *b'a'c'* of the polyhedron 2 on the Schlegel diagram corresponds to a CW direction around the corresponding face on the polychoron in four-dimensional space. The red lines on $D_1$ indicate the s-face, and the dashed one indicates the smallest-ID edge of the s-face. Similarly, the purple lines on the polyhedron 2 indicate the face $2_1$, and the dashed one indicates the edge $2_1$. $D_2$ is obtained by gluing the face $2_1$ of the polyhedron 2 to the s-face of $D_1$ in such a way that the edge $2_1$ is glued to the smallest-ID edge of the s-face.

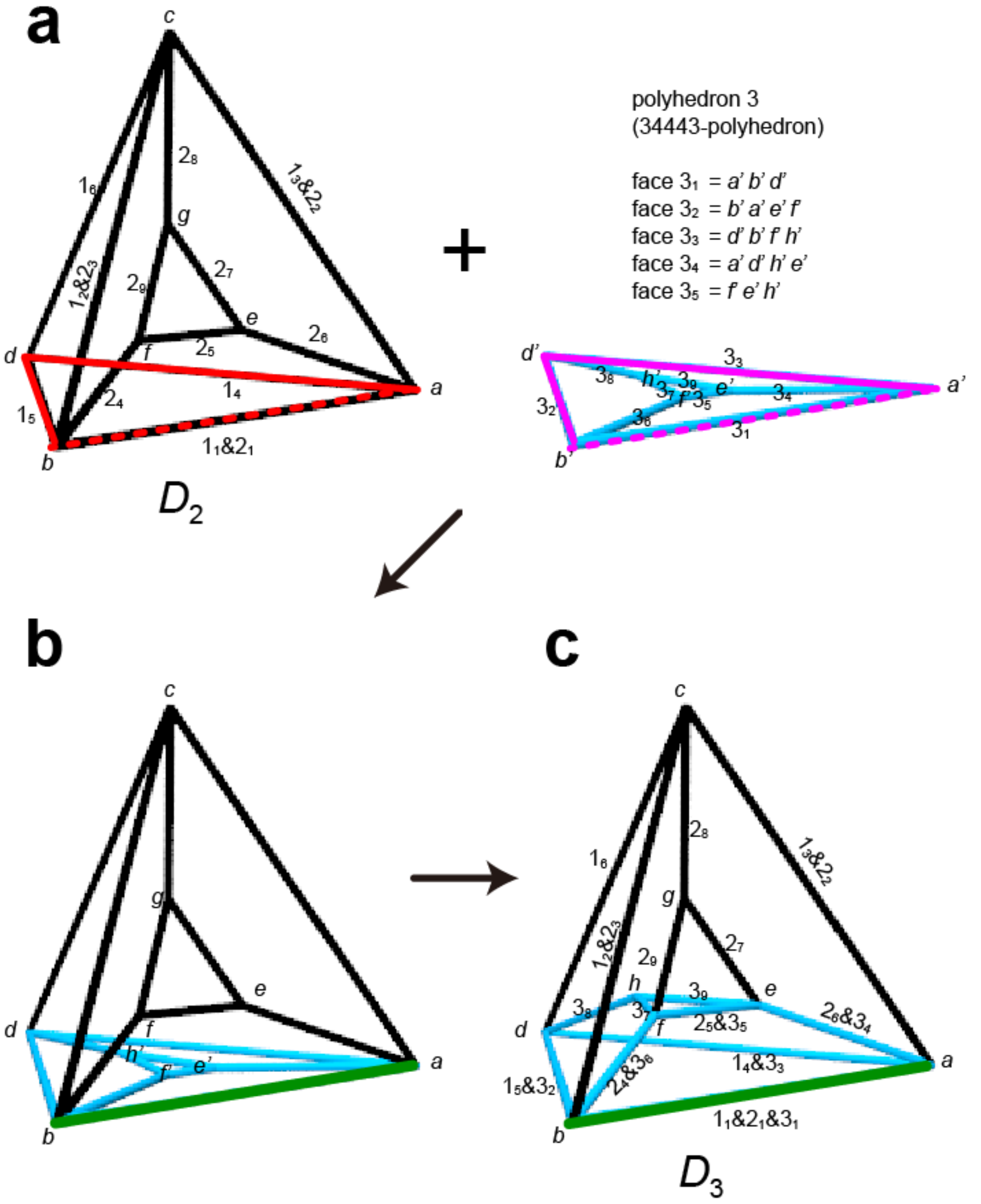


**Supplementary Figure S6 | How to decode 3333 34443 34443 34443 34443 3333 (Part 2). a**, The red lines on $D_2$ indicate the s-face, and the dashed one indicates the smallest-ID edge of the s-face. Similarly, the purple lines on the polyhedron 3 indicate the face $3_1$, and the dashed one indicates the edge $3_1$. **b**, To construct $D_3$, we first glue the face $3_1$ of the polyhedron 3 to the s-face of $D_2$ in such a way that the edge $3_1$ is glued to the smallest-ID edge of the s-face. Since the three polyhedra *abcd*, *bacfeg*, and *abde'f'h'* contribute to the peak *ab* coloured green that is also contributed by two dangling faces *abfe* and *abf'e'*, the peak *ab* is an i-peak. **c**, $D_3$ is obtained by rectifying the i-peak, specifically, by gluing together the two dangling faces *abfe* and *abf 'e'*.

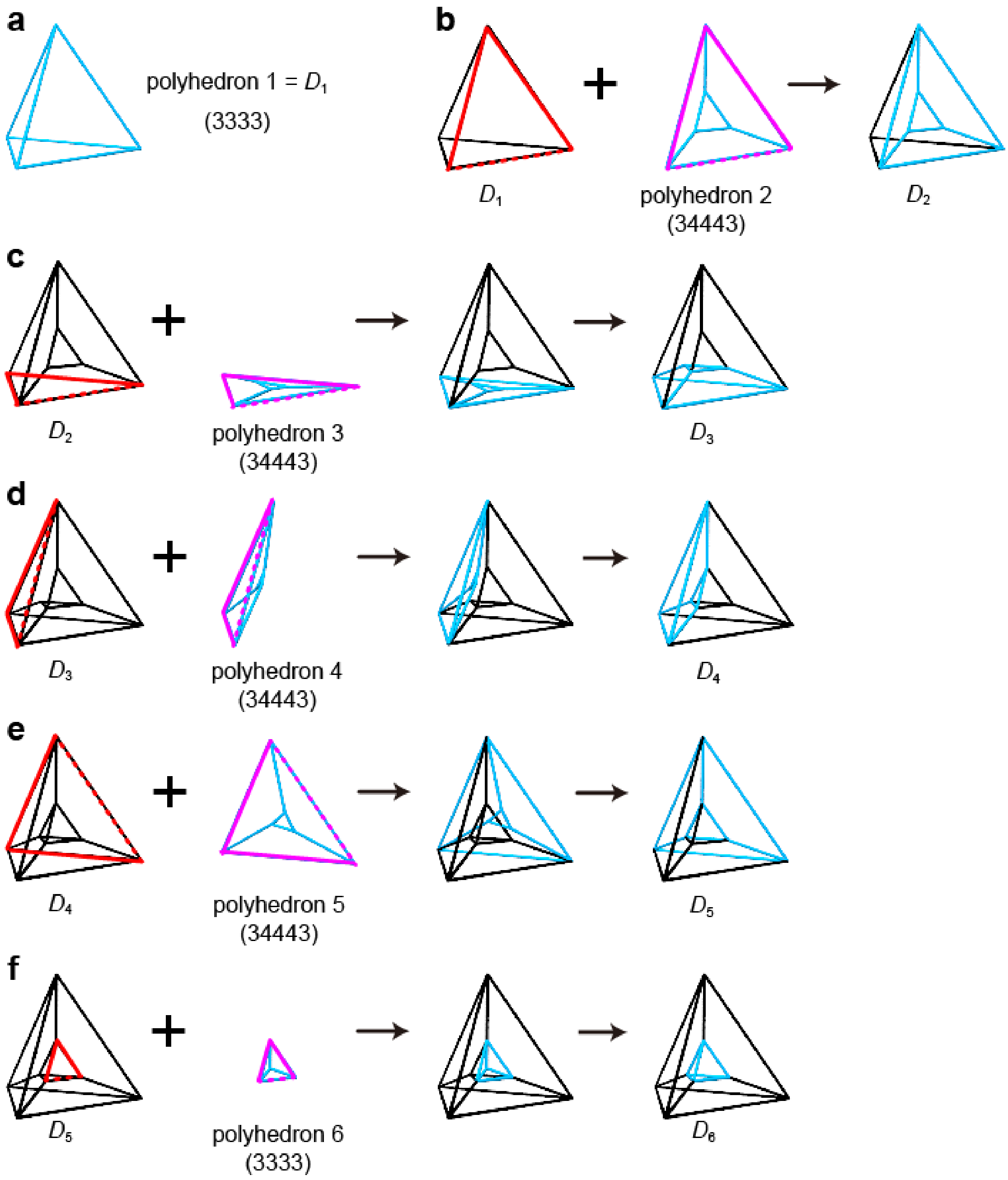


**Supplementary Figure S7 | How to decode 3333 34443 34443 34443 34443 3333 (Part 3).**

**Supplemental Note**

**Redundancy in *$ps_2$*-codeword.** We show that, if a polyhedron is simple, the last two digits $p_2(F\text{-}1)$ $p_2(F)$ in $ps_2$ are redundant. Specifically, $p_3$ can be deduced from a partial codeword $p_3^{(-2)}$ (= $p_2(1)$ $p_2(2)$ $p_2(3)$ ··· $p_2(F\text{-}2);sp$).

We first show that $p_2(F)$ is redundant. Consider a polyhedron that is encoded as $p_2(1)$ $p_2(2)$ $p_2(3)$ ··· $p_2(F);sp$, and suppose that we know the first ($F$-1) digits of $ps_2$, namely, $p_2(1)$ $p_2(2)$ $p_2(3)$ ··· $p_2(F\text{-}1)$, but we do not know the last digit $p_2(F)$. We then ask whether the original polyhedron can be recovered from the partial codeword $p_3^{(-1)}$ (= $p_2(1)$ $p_2(2)$ $p_2(3)$ ··· $p_2(F\text{-}1);sp$). To answer this question, we construct $D_{F\text{-}1}$ from $p_3^{(-1)}$. Since a complete polyhedron can be obtained by gluing a polygon to $D_{F\text{-}1}$, the partial polyhedron has only one closed plot (for example, $D_4$ of Supplementary Fig. S2a and $D_{10}$s of Supplementary Figs. S2b and S2c). Let $L(i)$ be the number of dangling sides in the s-plot of $D_i$. The polyhedron will be completed if and only if we glue an $L(F\text{-}1)$-gon to $D_{F\text{-}1}$ ($D_5$ of Supplementary Fig. S2a and $D_{11}$s of Supplementary Figs. S2b and S2c). We can thus determine $p_2(F)$ from $p_3^{(-1)}$ as $p_2(F) = L(F\text{-}1)$. We note that $p_2(F)$ can also be determined from a counting formula[22],

$$3c_3+2c_4+c_5-c_7-2c_8-3c_9-\cdots = 12.$$

Here, $c_i$ is the number of $i$-gons on the polyhedron.

We then show that the original polyhedron can be recovered from a partial codeword $p_3^{(-2)}$ (= $p_2(1)$ $p_2(2)$ $p_2(3)$ ··· $p_2(F\text{-}2);sp$). Since $p_2(F)$ can be determined from $D_{F\text{-}1}$, we illustrate how to determine $D_{F\text{-}1}$ from $p_3^{(-2)}$. To do this, we need to consider the two situations:

Case 1. Here, the polygons ($F$-1) and $F$ adjoin each other on the original polyhedron (for example, red and blue polygons on $D_5$ of Supplementary Fig. S2a). We can construct $D_{F\text{-}2}$ directly from $p_3^{(-2)}$. Since the polygons ($F$-1) and $F$ adjoin each other, $D_{F\text{-}2}$ must have only two plots adjoining each other that form a closed circle ($D_3$ of Supplementary Fig. S2a). The partial polyhedron with only one closed plot will be obtained if and only if we glue an ($L(F\text{-}2)$+1)-gon to $D_{F\text{-}2}$ ($D_4$ of Supplementary Fig. S2a). Therefore, $p_2(F\text{-}1)$ is determined as $p_2(F\text{-}1) = L(F\text{-}2)+1$.

Case 2. Here, the polygons ($F$-1) and $F$ are separate from each other on the original polyhedron (for example, red and blue polygons on $D_{11}$s of Supplementary Figs. S2b and S2c). When we construct $D_{F\text{-}2}$ from $p_3^{(-2)}$, two additional situations arise:

(I) If no missing pair remains, $D_{F\text{-}2}$ must have only two separate closed plots (for example, $D_9$ of Supplementary Fig. S2b). A partial polyhedron with a closed plot will be obtained if and only if $p_2(F\text{-}1) = L(F\text{-}2)$ ($D_{10}$ of Supplementary Fig. S2b).

(II) If a missing pair remains, $D_{F\text{-}2}$ must have at least four plots that form a closed circle (for example, $D_9$ of Supplementary Fig. S2c). The missing pair will be cured to obtain a partial polyhedron with a closed plot if and only if $p_2(F\text{-}1) = L(F\text{-}2)$ ($D_{10}$ of Supplementary Fig. S2c).

From the above discussions, $p_2(F\text{-}1)$ can be uniquely determined from $D_{F\text{-}2}$ as follows: If there remain only two plots that adjoin each other, $p_2(F\text{-}1) = L(F\text{-}2)+1$, otherwise, $p_2(F\text{-}1) = L(F\text{-}2)$.

Although $p_2(F\text{-}1)\ p_2(F)$ can be uniquely determined from $p_3^{(-2)}$, $p_2(F\text{-}2)$ cannot be determined from a partial codeword $p_3^{(-3)}$ (= $p_2(1)\ p_2(2)\ p_2(3) \cdots p_2(F\text{-}3);sp$). Actually, Supplementary Fig. S2d shows that different 448484433- and 448484334-polyhedra take the same $p_3^{(-3)}$-codeword (448484).

Altogether, every simple polyhedron can be represented without the last two digits of $ps_2$. However, we purposely admit this small redundancy in $ps_2$ to explicitly express all the information about the building-block polygons.

**Comparison with the previous methods for polyhedra.** The Schläfli symbol may be used to describe some of highly symmetric polytopes[12], but is not applicable to polytopes in general.

The index $c_3c_4c_5\cdots$ has been traditionally used to classify polyhedra in general[1-6,9,11], where $c_i$ is the number of $i$-gons on the polyhedron. However, polyhedra with different topologies could have the same index by chance, preventing a close characterization of polyhedral tilings[11].

The Weinberg code[11,13] is based on the traversal of an Euler path on a polyhedral graph. Its codeword consists of as many as $3V$+1 digits, where $V$ is the number of vertices on the polyhedron. For example, the representative Weinberg codeword for a trigonal prism is 1234145616265354321. On the other hand, our representative $p_3$-codeword for the same polyhedron is just 34443. Moreover, since the last two digits are extraneous, the first three digits 344 is enough.

The spiral code[14-16] is inapplicable to non-simple polyhedra, and is limited to simple ones. The representative spiral codeword for the polyhedron shown in Supplementary Fig. S2c is "6 3 8 3 8 3 8 3 6 0 24 46 3 3"[15]. Our $p_3$-codeword for the same polyhedron

is just 36883833633. Moreover, since the last two digits are redundant, the first nine digits 368838336 is enough.
